# Assessing colposcopic accuracy for high‐grade squamous intraepithelial lesion detection: a retrospective, cohort study

**DOI:** 10.1186/s12905-022-01592-6

**Published:** 2022-01-11

**Authors:** Anying Bai, Jiaxu Wang, Qing Li, Samuel Seery, Peng Xue, Yu Jiang

**Affiliations:** 1 Department of Epidemiology and Biostatistics, School of Population Medicine and Public Health, Chinese Academy of Medical Sciences and Peking Union Medical College, Beijing, 100730 China; 2 Diagnosis and Treatment for Cervical Lesions Center, Shenzhen Maternity & Child Healthcare Hospital, Shenzhen, 518028 China; 3 Division of Health Research, Faculty of Health and Medicine, Lancaster University, Lancaster, LA1 4YW UK

**Keywords:** Cervical cancer, Colposcopy, Cervical biopsy, High-grade squamous intraepithelial lesion

## Abstract

**Background:**

Inappropriate management of high-grade squamous intraepithelial lesions (HSIL) may be the result of an inaccurate colposcopic diagnosis. The aim of this study was to assess colposcopic performance in identifying HSIL+ cases and to analyze the associated clinical factors.

**Methods:**

Records from 1130 patients admitted to Shenzhen Maternal and Child Healthcare Hospital from 12th January, 2018 up until 30th December, 2018 were retrospectively collected, and included demographics, cytological results, HPV status, transformation zone type, number of cervical biopsy sites, colposcopists’ competencies, colposcopic impressions, as well as histopathological results. Colposcopy was carried out using 2011 colposcopic terminology from the International Federation of Cervical Pathology and Colposcopy. Logistic regression modelling was implemented for uni- and multivariate analyses. A forward stepwise approach was adopted in order to identify variables associated with colposcopic accuracy. Histopathologic results were taken as the comparative gold standard.

**Results:**

Data from 1130 patient records were collated and analyzed. Colposcopy was 69.7% accurate in identifying HSIL+ cases. Positive predictive value, negative predictive value, sensitivity and specificity of detecting HSIL or more (HSIL+) were 35.53%, 64.47%, 42.35% and 77.60%, respectively. Multivariate analysis highlighted the number of biopsies, cytology, and transformation zone type as independent factors. Age and HPV subtype did not appear to statistically correlate with high-grade lesion/carcinoma.

**Conclusion:**

Evidence presented here suggests that colposcopy is only 69.7% accurate at diagnosing HSIL. Even though not all HSIL will progress into cancer it is considered pre-cancerous and therefore early identification will save lives. The number of biopsies, cytology and transformation zone type appear to be predictors of misdiagnosis and therefore should be considered during clinical consultations and by way of further research.

## Background

Cervical cancer is one of the most prevalent genital tumors and poses a threat to every women’s health [1]. In 2020, cervical cancer caused approximately 340,000 deaths with a further 600,000 new cases recorded. This accounts 3.4% of all deaths and 3.3% of all cancers incidents, globally. In China alone, cervical cancer is of growing concern [2], where according to national cancer statistics, the incidence of cervical cancer in 2015 was 9.89 per 10,000 with a 3.05 per 10,000 mortality rate [3]. Human papillomavirus (HPV) infection is universally recognized as a causative agent in the development of cervical intraepithelial neoplasia (CIN) and squamous intraepithelial lesions. These can be benign but are considered pre-cancerous and often develop into invasive cervical carcinoma [4]. As precursors to invasive squamous carcinoma, over one third of all high-grade squamous intraepithelial lesions (HSIL) and CIN grades II and III, progress into cervical cancer over a period of between 10 and 15 years [5].

In order to combat the global prevalence and mortality of cervical cancer most countries have implemented screening programs which utilize cervical cytology and/or HPV testing, and then colposcopy when screening finds abnormal cells in the cervix. Therefore, colposcopy is an indispensable tool for early detection with accurate use benefiting women by reducing the number of unnecessary biopsies, conization procedures, as well as the frequency of cauterization therapy for cervical erosions [6]. This means, there is a great deal of avoidable stress caused by diagnostic inaccuracies and discrepancies between colposcopic and pathologic diagnosis are known [7]. Colposcopy is considered a subjective procedure, which is dependent upon a clinician’s assessment. Many factors can therefore bias diagnosis, such as knowing cytologic results, or HPV subtypes and transformation zone types. Therefore, it remains necessary to identify reasons for diagnostic inaccuracies, in order to reduce unnecessary stress caused and improve outcomes.

In 2011, the International Federation of Cervical Pathology and Colposcopy (IFCPC) provided a new terminology system based on versions developed in 1975 [8], 1990 [9]. and 2002 [10]. This new system provides a more comprehensive understanding with evidence-based reclassifications of some abnormal colposcopic findings [6]. However, there are few studies which evaluate the 2011 IFCPC terminology and despite the prevalence of cervical cancer in China there are very few which utilize the IFCPC system across a Chinese sample. Therefore, it is not only important to identify and assess potential causes of colposcopic inaccuracies but also to understand diversity and variance. The primary aim of this study was to assess levels of agreement between colposcopy and cervical biopsy in identifying HSIL. However, this also necessitates an investigation into the associated clinicopathological factors affecting diagnostic accuracy.

## Methods

### Study population

Patient records for those who underwent colposcopic examination at Shenzhen Maternal and Child Health Hospital from 12th January 2018 to 30th December 2018 were retrospectively analyzed. Patients without adequate colposcopy impressions, HPV, cytology or histopathological results, and those who did not have basic demographic information, were excluded. The final sample consisted of 1130 patients.

Demographics and clinical characteristics including age, cytological examination results, HPV status, transformation zone type, number of cervical biopsy sites, colposcopy results and pathological results, were collected. This study was conducted ethically in accordance with the World Medical Association Declaration of Helsinki. This study was approved by the institutional review board (IRB) of Shenzhen maternal and Child Health Hospital (No. 164), and the need for informed consent was waived by the IRB of SZMCHH due to the retrospective nature of archived datasets and fully anonymized personal information.

### HPV and cytology subtypes/categories

HPV genotyping was detected in liquid-based cytology specimens collected using the HPV GenoArray test kit (HybriBio Ltd). This kit is capable of identifying 15 high-risk HPV (hrHPV) types (16, 18, 31, 33, 35, 39, 45, 51, 52, 56, 58, 59, 65, 66, and 68) [11]. In this study, patients were categorized as HPV negative, HPV 16/18 and other HR-HPV positive type.

Thinprep cytologic test (TCT, Hologic, USA) were used to perform cytologic analysis. Results were reported according to the Bethesda System [12] and categorized into five classes: negative for intraepithelial lesion or malignancy (NILM), atypical squamous cells of undetermined significance (ASCUS), atypical squamous cells—cannot exclude high-grade squamous intraepithelial lesion (ASC-H), low-grade squamous intraepithelial lesion (LSIL), and HSIL and/or squamous cell carcinoma (HSIL/SCC).

### Colposcopy and biopsy

General assessment was conducted in accordance with the 2011 IFCPC colposcopic terminology for the cervix [13] which includes transformation zone types 1, 2 or 3. All colposcopies were performed by gynecologists using an electronic colposcope (Goldway Ltd). The number of biopsies was reported with histopathologies, and pathologic results were taken as the gold standard.

Patients with confirmatory colposcopic and pathologic results for HSIL+ or < HSIL were categorised into the ‘concordant’ group. All others were assigned to the group labelled ‘discordant’. The accordance rate is the percentage of patients with confirmatory colposcopic and histopathologic findings. Overdiagnosis was considered present when histopathologic results highlighted less advanced lesions than colposcopic examination. Underdiagnosis was considered to have occurred when histopathological results highlighted more advanced lesion/s than colposcopic examination.

### Statistical analysis

The characteristics of the concordant and disconcordant groups were compared using Pearson’s χ^2^ test for qualitative variables and Student’s t-test for quantitative variables. Differences between underdiagnosis, overdiagnosis and accordance between subgroups of all the patients were examined using χ^2^ tests or Fisher’s exact tests.

Logistic regression modelling was used for uni- and multivariate analyses. A forward stepwise approach was implemented to identify variables influencing colposcopic accuracy.

All *p* values are two-sided, with the threshold for statistical significance set at 0.05. All statistical analyses were performed using SAS software (version 9.4). All methods were performed in accordance with the Declaration of Helsinki.

## Results

### Clinical characteristics of study population

Table 1 provides summaries of clinical characteristics for all patients and the results of subgroup analysis including age of the women at the time of examination, cytologic results, HPV genotype, and transformation zone information. Of the 1130 patients included in this study, 69.64% (n = 787) received a consistent diagnosis, whereas 30.35% (n = 343) of all HSIL+ patients were not identified through colposcopy. The diagnostic accuracy of HSIL+ cases correlated positively with increasing age. That is, we observed 62.26% accuracy for those below 30 years, 71.64% for those aged between 30 and 45, and 69.42% for age 45 or older (*p* = 0.031).

**Table 1:** Characteristics of all patients for subgroup analysis

Variables	Discordant	Concordant	*p*
	n = 343	n = 787	
*Age categories*			0.031
< 30	37.74% (n = 80)	62.26% (n = 132)	
30–45	28.36% (n = 226)	71.64% (n = 571)	
> 45	30.58% (n = 37)	69.42% (n = 84)	
*Cytology results*			< 0.001
NILM	24.34% (n = 176)	75.66% (n = 547)	
ASC-US	42.44% (n = 101)	57.56% (n = 137)	
LSIL	39.58% (n = 57)	60.42% (n = 87)	
ASC-H	66.67% (n = 4)	33.33% (n = 2)	
HSIL	26.32% n = 5)	73.68% (n = 14)	
*HPV subtypes*			0.001
Negative	30.59% (n = 26)	69.41% (n = 59)	
Other HR-HPV positive	37.91% (n = 127)	62.09% (n = 208)	
HPV16/18	26.76% (n = 190)	73.24% (n = 520)	
*Transformation zone type*			< 0.001
TZ 1	20.20% (n = 61)	79.80% (n = 241)	
TZ 2	32.56% (n = 42)	67.44% (n = 87)	
TZ 3	34.33% (n = 240)	65.67% (n = 459)	
Number of biopsies	1.1603 ± 2.1180	1.4727 ± 1.5920	0.006

Only 73.7% of all HSIL cases were detected through colposcopy. Cytologic analysis found consistently benign results across subgroups, as follows: NILM (75.66%), ASC-US (57.56%), LSIL (60.42%), and ASC-H (33.33%). When detecting HSIL+ cases there was a significant difference across transformation zones (*p* < 0.001) with TZ 1 associated with the highest diagnostic accuracy (79.80%), TZ 2 (67.44%) and TZ 3 (65.67%).

The consistent rate in patients with HPV 16 or 18 genotypes was 73.24%, which was higher than other HPV positive genotypes, but marginally lower among patients who tested negative for HPV (*p* = 0.001).

Tables 2, 3, 4 and 5 provide results of subgroup analysis i.e. age of the women at the time of examination, cytology results, HPV subtypes and transformation zone types. The accuracy for HSIL+ detection decreased with increasing age, and HSIL+ cases were more likely to be missed among older women (please see Table 2. for details). At the same time, the chances of underdiagnosing and overdiagnosing decreased among HSIL patients more than patients with other subtypes (*p* < 0.001). See Table 3 for further comparisons.

**Table 2:** Accordance rate, over- and underdiagnosis of HSIL+ relative to patients’ age (*p* = 0.190)

	0–30 years (n = 268)	31–45 years (n = 741)	> 45 years (n = 139)	Total
Overdiagnosis	19.78% (n = 53)	17.14% (n = 127)	11.51% (n = 16)	196
Accordance	65.67% n = 176)	71.12% (n = 527)	60.43% (n = 84)	787
Underdiagnosis	14.55% (n = 39)	11.74% (n = 87)	15.11% (n = 21)	147

**Table 3:** Accordance rate, over- and underdiagnosis of HSIL+ relative to cytologic results (*p* < 0.001)

	NILM (n = 723)	ASC-US (n = 238)	LSIL (n = 144)	ASC-H (n = 6)	HSIL (N = 19)
Overdiagnosis	21.72% (n = 157)	9.66% (n = 23)	8.33% (n = 12)	33.33% (n = 2)	10.53% (n = 2)
Accordance	75.66% (n = 547)	57.56% (n = 137)	60.42% (n = 87)	33.33% (n = 2)	73.68% (n = 14)
Underdiagnosis	2.63% (n = 19)	32.77% (n = 78)	31.25% (n = 45)	33.33% (n = 2)	15.79% (n = 3)

**Table 4:** Accordance rate, over- and underdiagnosis of HSIL+ relative to HPV subtypes (*p* < 0.001)

	Negative	HPV16/18	Other HR-HPV positive
Overdiagnosis	24.71% (n = 21)	13.66% (n = 97)	23.28% (n = 78)
Accordance	69.41% (n = 59)	73.42% (n = 520)	62.09% (n = 208)
Underdiagnosis	5.88% (n = 5))	13.10% (n = 93)	14.63% (n = 49)

**Table 5:** Accordance rate, over- and underdiagnosis of HSIL+ relative to transformation zone type (*p* < 0.001)

	TZ 1 (N = 302)	TZ 2 (N = 129)	TZ 3 (N = 699)	Total
Overdiagnosis	12.91% (n = 39)	14.73% (n = 19)	19.74% (n = 138)	196
Accordance	79.88% (n = 241)	67.44% (n = 87)	65.67% (n = 459)	787
Underdiagnosis	7.28% (n = 22)	17.83% (n = 23)	14.59% (n = 102)	147

The accordance rate between colposcopic and pathologic examinations was highest among HPV 16/18 cases. While the rate of overdiagnosis was worse in negative patients, the rate of underdiagnosis was highest among patients with other HR-HPV positive results (Table 4). Differences in detection accuracy among HSIL+ patients with TZ 1, TZ 2, or TZ 3 were significant (*p* < 0.001). The rate of overdiagnosis was worse in women with TZ 3 (19.74%), whereas women with TZ 1 or TZ 2 appear to be overdiagnosed with 12.91% and 14.73%, respectively (Table 5).

Table 6 provides summaries of the results of multivariate logistic regression. The number of cervical biopsy sites, age group, cytological results and transformation zone types appear as significant influences over colposcopic accuracy. Age of patients and HPV subtypes do not appear to be related (*p* = 0.504).

**Table 6:** Logistic regression analysis of factors influencing colposcopic accuracy in detecting HSIL+ (n = 1130)

Variables	β	OR (95% CI)
*Cytology results*		
NILM		1.000
ASC-US	− 0.8060	0.447 (0.325–0.613)
LSIL	− 0.7344	0.480 (0.326–0.706)
ASC-H	− 1.6004	0.202 (0.036–1.143)
HSIL	− 0.3302	0.719 (0.250–2.067)
*HPV*		
Negative		1.000
Other HR-HPV positive	− 0.1801	0.835 (0.492–1.417)
HPV16/18	0.2455	1.278 (0.771–2.119)
*Transformation zone type*		
TZ 1		1.000
TZ 2	− 0.5993	0.549 (0.342–0.883)
TZ 3	− 0.6522	0.521 (0.375–0.724)
Number of biopsies	0.1355	1.145 (1.052–1.246)

A greater the number of cervical biopsy sites appears to positively correlate with higher odds of accurate detection (OR 1.15, 95% CI 1.05–1.25). Compared with NILM, patients whose cytologic results were ASC-US (OR 0.45, 95% CI 0.325–0.613) or LSIL (OR 0.48, 95% CI 0.326–0.706) were significantly associated with decreased odds of detection accordance. Patients with TZ 2 (OR 0.55, 95% CI 0.34–0.88) and TZ 3 (OR 0.52, 95% CI 0.38–0.72) significantly correlated with decreased odds of detection accuracy, compared with TZ 1.

Table 7 presents the results of associations between patient characteristics and the accuracy of colposcopy in detecting HSIL+. We measured the detection of HSIL+ directly, according to an increasing number of lesion-directed biopsies, separately for women with one, two and three or more biopsies. The accuracy of colposcopy significantly associated with numbers of biopsies among patients whose cytologic results ≤ NILM or those who were HPV negative or with other HR-HPV positive. Among all patients, the first biopsy increased from 52.87% in women with one directed biopsy to 79.44% in women with two lesion-directed biopsies (*p* = 0.027), reflecting the increasing severity of the cases. However, a third or more biopsies decreased the accuracy of cytology by 2.79% compared with two biopsies. By contrast, the accuracy for the first biopsy consistently decreased from 92.31% to 64.13% in women with three or more lesion-directed biopsies among patients with HPV negative or positive with other HR-HPV type (*p* = 0.004), and decreased from 89.74% to 79.80% among patients with cytology results ≤ NILM (*p* = 0.036).

**Table 7:** Associations between patient characteristics and the accuracy of colposcopy in detecting HSIL+ with increasing number of lesion-directed biopsies (n = 1130)

Variables	Discordant	Concordant	P
All (n = 1130)			0.0265
*Number of biospies*			
1	47.13% (n = 41)	52.87% (n = 46)	
2	20.56% (n = 59)	79.44% (n = 228)	
≥ 3	25.00% (n = 63)	75.00% (n = 189)	
Cytology ≤ NILM (n = 484)			0.0362
*Number of biospies*			
1	10.26% (n = 4)	89.74% (n = 35)	
2	17.41% (n = 43)	82.59% (n = 204)	
≥ 3	20.20% (n = 40)	79.80% (n = 158)	
Cytology ≥ ASC-US (n = 101)			0.4779
*Number of biospies*			
1	14.29% (n = 1)	85.71% (n = 6)	
2	40.00% (n = 16)	60.00% (n = 24)	
≥ 3	42.59% (n = 23)	57.41% (n = 31)	
HPV-/Other HR-HPV + (n = 190)			0.0038
*Number of biospies*			
1	7.69% (n = 1)	92.31% (n = 12)	
2	17.65% (n = 15)	82.35% (n = 70)	
≥ 3	35.87% (n = 33)	64.13% (n = 59)	
HPV 16/18 (n = 395)			0.4263
*Number of biospies*			
1	12.12% (n = 4)	87.88% (n = 29)	
2	21.78% (n = 44)	78.22% (n = 158)	
≥ 3	18.75% (n = 30)	81.25% (n = 130)	

## Discussion

Cervical intraepithelial neoplasia and squamous intraepithelial lesions are considered pre-cancerous and often develop into invasive cervical carcinoma within 15 years of the original diagnosis. Unlike many conditions there is an opportunity for early diagnosis which improves outcomes, dramatically. However, neither screening nor later colposcopic examination are perfectly accurate for every individual, which means that women are often misdiagnosed or unnecessarily scared and embarrassed by unnecessary biopsies. Therefore, it is necessary to ensure we understand the factors which influence colposcopy. As such, an aim of this study was to assess agreement between colposcopy and cervical biopsy in identifying HSIL. Associated clinicopathological factors affecting diagnostic accuracy were then analyzed for research and development.

Records from 1130 patients admitted to SZMCHH from January up until 30^th^ December, 2018 were collected. Demographics and clinical characteristics including age, cytological examination results, HPV status, transformation zone type, number of cervical biopsy sites, colposcopy results and pathological results, were analyzed. The overall diagnostic accuracy of colposcopy in identifying HSIL+ was 69.64% which appears low. However, colposcopic accuracy has always been questioned because agreement between colposcopic diagnosis and cervical biopsy analysis varies between countries and even between hospitals. In this study, the sensitivity of colposcopic examination for detecting HSIL+ was 42.35%, and the specificity was 77.60%, which were similar to previous studies conducted in other cities of China [6, 14, 15].

Agreement between colposcopic diagnosis and final pathology matched in 69.64% of cases in this study, which was also comparable to previous research in China. For example, Li et al. found agreement between colposcopic impression and histopathological diagnosis was 46.9% [15] using 2011 IFCPC colposcopic terminology, with a sensitivity for colposcopic examination at detecting HSIL+ at 54.7%. Importantly, even though the Li et al. study was conducted in western China, there are differences which may not be based entirely upon sample size differences. As has been mentioned, colposcopic impressions are often biased by knowledge of a patient’s history and previous tests administered. It was found by way of a systematic review that the positive predictive value for HSIL in the diagnosis of CIN2+ worldwide is 77.5% [16]. Ouitrakul et al. reported that the accuracy of colposcopically directed biopsy to detect HSIL or more of the uterine cervix was 87.8% sensitive [17]. This shows that the diagnostic value of cytological HSIL results in the diagnosis of CIN2+ lesions is reasonable yet this is not high enough to rely solely on cytology for cervical lesions.

These inaccuracies have prompted some to recommend a combination of tests, or serial co-testing, as this could improve accuracy; however, unnecessary testing impacts upon both the affordability of healthcare and individuals’ psychological well-being. It should be noted that differences may also be due to the use of colposcopic terminology, as well as heterogeneous sample characteristics, and the level and experience of colposcopists. For example, in a large clinical study conducted in Australia with 18,421 satisfactory colposcopies performed between 1999 and 2004, researchers found that colposcopy was 60% sensitive and with 60% PPV in identifying HSIL [18]. However, the PPVs in detecting HSIL [19] across included studies in a meta-analysis varied between 20 and 84% [19]. This highlights issues which occur when synthesizing secondary retrospective data and does not provide reasons for such variability. Additionally, previous studies have emphasized specific, compulsory training for trainees before becoming qualified colposcopists, especially in low and middle-income countries [18].

We found a number of variables which are likely to influence colposcopic accuracy. In order to identify significant variables, we assessed colposcopic accuracy compared with final diagnosis in relation to different subgroups. We found that even though diagnostic accuracy of HSIL+ appeared to positively relate to increasing age in different patient groups, multivariate logistic regression analysis did not suggest age is an independent predictor factor. The reason for this might be heterogeneity within the sample or specific patient characteristics including TZ types and cytologic results. However, several clinical trials have reported that the risk of HSIL+ actually decreases with increasing age among HPV + women under 40 years of age with negative cytology [20, 21]. This, the authors suggested, occurred because of age-related biological changes in the cervical transformation zone making the cervix less susceptible to new infection, or perhaps making small lesions more difficult to detect with a colposcope.

Some studies have also observed that the diagnostic accuracy of colposcopy-directed biopsy for identifying HSIL+ decreased as age increased. For example, Kim et al. [22] reported that the balanced accuracy of colposcopy-directed biopsy was 81% for those < 35 years, 74.4% for 35–50 years, and 68.8% for those older than 50 years. These findings highlight a decline in balanced accuracy with increasing age which Stuebs et al. [23] also found when studying accuracy rates for detecting HSILs using 2011 IFCPC colposcopic terminology, which were 93.1% (age 0–34), 83.6% (age 34–55), and 80% (age 55 or older). The authors themselves postulated that relatively poor diagnostic performances at identifying HSIL+ cases in those ≥ 50 years of age might be related to menopause and to unidentifiable squamocolumnar junctions or cervical lesions with limits that are not easily visualized using colposcopy although, this requires further research.

The number of cervical biopsy sites and cytologic results appeared in this study to be significantly related to colposcopic accuracy. We found an increasing number of cervical biopsy sites was significant, with 1.15 times higher odds of accurate detection. Gage et al. found that the sensitivity of enrollment colposcopic procedure did not vary significantly according to colposcopist’s professional characteristics but that sensitivity was significantly greater when colposcopists took two or more biopsies [24], This finding has been confirmed by a study by Robert et al. [25] and Wentzensen et al. [26] who reported the highest sensitivity for detecting high-grade dysplasia was 95.6% after taking three biopsies according to the standards of the American Society for Colposcopy and Cervical Pathology (ASCCP). Although, it is not always the case that three of more biopsies will further improve identification. Zuchna et al. found that sensitivity increased after taking a second biopsy, but that there was no further improvement after taking a third biopsy [27].

Unlike previous research, we further found that the optimal number of biopsies depends on prior risk, as determined by cytology status, colposcopy impression, and HPV type status. Though the incremental benefit of taking two biopsies was present compared with only one site among all patients, our results suggested limited benefit from additional biopsies among patients with cytology results ≤ NILM or negative and other positive HPV types, which might even lead to the overdiagnosis of detecting HSIL+ cases using colposcopy. Our results were in accordance with previous findings [28]. Therefore, ‘second-look’ biopsies and perhaps even a third biopsy may be necessary to ensure we do not miss opportunities to intervene, and future study using larger sample sizes might be conducted to improve the performance of colposcopy based on stratified patient risks.

In this study, we found that 75.7% (NILM), 57.6% (ASC-US), 60.4% (LSIL) and 33.3% (ASC-H) of cases with different cytology results had benign conclusions through colposcopic and pathologic examinations, with an accuracy rate of 73.7% in HSIL cases. The accuracy of colposcopy-directed biopsy was however lower for detecting LSIL when compared with HSIL, which was in line with previous studies. Tatiyachonwiphut et al. [29] found in colposcopic diagnosis of high-grade lesions, that pathologic determination will be either HSIL, MIC or invasive cancer in 75.5% of the study population. Conversely, in colposcopic diagnosis is low grade lesions, the cervical pathology will be normal, benign or LSIL in 83.8%. A prospective multicenter trial in Austria reported that the sensitivity of colposcopically directed cervical biopsies was 66.2% [27]. Specifically, the agreement between histological results on biopsy and cone specimen was 54.5% for low-grade lesions, 78.2% for high-grade lesions, and 28.9% for microinvasive cervical cancer [30]. Similarly, Howe and Vincenti [31] and Zuchna et al. [27] found that detection rates among LSIL women were around 31%, while Baldauf et al. [32] reported an accuracy rate of over 80% for LSIL cases, which suggests there is a great degree of variability which demands further attention.

Loiudice et al. [33] found that agreement between LSIL and histologic results was 37%, and agreement between HSIL and histologic results was 76% which appears incongruous with our findings. Our results suggest that 31.25% of all LSIL cases were underdiagnosed, yet only 8.33% were overdiagnosed. These findings were consistent across ASC-US and HSIL subgroups, and generally in line with the assertion that colposcopic impressions are more likely to overestimate rather than underestimate disease [7, 29]. This maybe because various lesion points are inadvertently removed during biopsy, or may occur because practitioners had knowledge of the cytology results before colposcopy. Again, these not-so-subtle differences appear to relate to a colposcopists’ skillsets or bias caused by assuming the biopsy is a form of colposcopic confirmation. The disagreement might be improved by advocating novel methods such as artificial intelligence used for grading colposcopic impressions and guiding biopsies [34, 35], as well as portable devices that can be used to perform colposcopy [36]. Both have shown potential in improving the diagnostic quality of colposcopy.

Among HPV-positive and HPV-negative patients, a higher percentage of overdiagnosis was observed, with the difference highly significant in HPV-negative patients. Zaal et al. [37] found a relatively high percentage of HPV- women with high-grade lesions i.e. 22.8%, which might be caused by false-negative HPV test results. Our study also found that among HPV + patients, the likelihood of underdiagnosis was higher, which was in line with previous studies [15]. However, the association between HPV subtypes and the accuracy of detecting HSIL+ using colposcopy was no longer significant under multivariate logistic regression. This suggests that the effect of HPV might diminish when assessed with other variables. Our study was not designed to determine whether different methods of HPV testing would bias colposcopic impressions although, it would seem prudent to investigate this further.

The 2011 IFCPC colposcopic terminology confirmed the classification of TZs as an obligatory terminology [6]. We found that the accuracy of detecting HSIL+ in women decreased from TZ 1 to TZ 3 (TZ 1, 79.8%; TZ 2, 67.4%; TZ 3, 65.7%). This appears to be in accordance with previous findings [23, 30] which was to be expected due to the classifications themselves. The components of TZ 1 mean that the entire transformation zone is visible, whereas in TZ 3 the entire upper limit may not be visible and therefore can be misleading [23]. From a specialist’s perspective, it seems necessary to focus further research on TZ 3 cases specifically because of the related diagnostic complexities. It appears the greatest number of false negative cases occur within this classification and therefore clinicians need additional indicators which might initiate a second-look biopsy or indeed a third where necessary. This is however, a fine balance between administering ‘unnecessary testing’ and managing patients’ anxieties and well-being.

To the best of our knowledge, this is the first study to assess the diagnostic accuracy of colposcopy in identifying HSIL+ among Chinese patients in Shenzhen, China. Populations in Chinese cities may be considered by some to be homogeneous but this is not the case and we also must look to understand potential differences between nationalities and ethnicities. Therefore, even though this study included only Chinese women it is necessary to develop our understanding of this population to make more reliable comparisons. This study was however retrospective with limited data availability which constrained our analysis. We tried to investigate the influence of colposcopists skills and experience although this was not included because we were unable to extract sufficient data to create subgroups. Of course, the relatively small sample sizes attributed to subgroups probably also affected our analysis of HPV subtypes, for example. The last major concern was that biopsy specimens were taken only from suspicious lesions without comparable control specimens.

## Conclusion

The overall diagnostic accuracy of colposcopy and the consistency between colposcopy and histopathology in our study were comparable to previous studies, but there is room for improvement. The number of biopsies, cytology and transformation zone type appear to be predictors of misdiagnosis and therefore should be considered more carefully during clinical consultations and by way of further research. Future colposcopy-based studies using a reasonable scoring system and standard diagnostic criteria are still urgently needed to assess colposcopists’ and colposcopic performance more objectively.

## Data Availability

The datasets used and/or analyzed during the current study are available from the corresponding author on reasonable request.
